# Publisher Correction: Fusion of multi-scale bag of deep visual words features of chest X-ray images to detect COVID-19 infection

**DOI:** 10.1038/s41598-022-05240-9

**Published:** 2022-01-13

**Authors:** Chiranjibi Sitaula, Tej Bahadur Shahi, Sunil Aryal, Faezeh Marzbanrad

**Affiliations:** 1 Department of Electrical and Computer Systems Engineering, Monash University, Clayton, VIC 3800 Australia; 2 School of Engineering and Technology, Central Queensland University, Rockhampton, QLD 4701 Australia; 3 Central Department of Computer Science and IT, Tribhuvan University, Kathmandu, 44600 Nepal; 4 School of Information Technology, Deakin University, Waurn Ponds, VIC 3216 Australia

Correction to: *Scientific Reports* 10.1038/s41598-021-03287-8, published online 13 December 2021

The original version of this Article contained errors in the affiliations, where Tej Bahadur Shahi was incorrectly affiliated with ‘School of Information Technology, Deakin University, Waurn Ponds, VIC 3216, Australia’, and Sunil Aryal was incorrectly affiliated with ‘Central Department of Computer Science and IT, Tribhuvan University, Kathmandu, 44600, Nepal’.

The correct affiliations are listed below:

Affiliation 3:

Central Department of Computer Science and IT, Tribhuvan University, Kathmandu 44600, Nepal

Tej Bahadur Shahi

Affiliation 4:

School of Information Technology, Deakin University, Waurn Ponds, VIC 3216, Australia.

Sunil Aryal

As a result, the affiliations have been renumbered.

The original Article has been corrected.

